# Axisymmetric Contact Problem for a Flattened Cell: Contributions of Substrate Effect and Cell Thickness to the Determination of Viscoelastic Properties by Using AFM Indentation

**DOI:** 10.1155/2017/8519539

**Published:** 2017-12-20

**Authors:** Xinyao Zhu, Lanjiao Liu, Zuobin Wang, X. Liu

**Affiliations:** ^1^School of Engineering, University of Warwick, Coventry CV4 7AL, UK; ^2^International Research Centre for Nano Handling and Manufacturing of China, Changchun University of Science and Technology, Changchun 130022, China

## Abstract

Nanoindentation technology has proven to be an effective method to investigate the viscoelastic properties of biological cells. The experimental data obtained by nanoindentation are frequently interpreted by Hertz contact model. However, in order to validate Hertz contact model, some studies assume that cells have infinite thickness which does not necessarily represent the real situation. In this study, a rigorous contact model based upon linear elasticity is developed for the interpretation of indentation tests of flattened cells. The cell, normally bonded to the Petri dish, is initially treated as an elastic layer of finite thickness perfectly fixed to a rigid substrate. The theory of linear elasticity is utilized to solve this contact issue and then the solutions are extended to viscoelastic situation which is regarded as a good indicator for mechanical properties of biological cells. To test the present model, AFM-based creep test has been conducted on living human hepatocellular carcinoma cell (SMMC-7721 cell) and its fullerenol-treated counterpart. The results indicate that the present model could not only describe very well the creep behavior of SMMC-7721 cells, but also curb overestimation of the mechanical properties due to substrate effect.

## 1. Introduction

The measurement of viscoelastic properties of living cells can provide important information about the biomechanical effects of drug treatment, diseases, and aging. To date, a variety of testing techniques have been used to measure the viscoelastic mechanical properties of biological cells, for example, micropipette aspiration [1], atomic force microscopy [2], optical tweezers [3, 4], and magnetic tweezers [5]. Compared with other techniques, AFM has many advantages such as direct interaction with the sample, flexibility in option of probe type, and convenient imaging of surface topography of cell. However, AFM-based quantification of the biomechanical property requires an appropriate rheological model which could describe the factual situation of cell indentation. Although Hertz contact model is frequently used to interpret the experimental data obtained by AFM indentation, one of its main assumptions, that is, treating indented cell as semi-infinite space, might be contradicted by the film morphology of cells after being removed from their native environment [6, 7]. In this sense, the estimation of cell properties would be affected by the stiff substrate. If not accounted for, substrate effect would lead to overestimation of the measured parameters [8–10], for example, elastic modulus, viscosity, and diffusion. In this regard, it is essential to develop an effective means to characterize the effect of film thickness in cell indentation.

The indentation of thin layer by spherical indenters has been commonly studied in the literature using either cumbersome numerical calculations or analytical modeling [11–14]. In this sense, Dimitriadis et al. [15] adopted an imaging method to present a convenient correction to Hertz model for thin and elastic film subject to spherical tip indentation. Based on this modification, AFM measurements with spherical tip become a common experimental method to quantify the mechanical properties of spread cells [6, 16]. With a spherical indenter, the measured mechanical properties only represent an average response of a sample, while information of features smaller than size of indenter would be missing [8]. In addition, although Dimitriadis's model has been modified to characterize conical tip indentation [8, 9], there exist inconsistency and nonuniformity between the multiplicative correction factors provided by these studies. In this regard, it is imperative to develop a universal correction to Hertz contact model to account for indentation of thin layers, regardless of size or profile type of the indenter.

In this work, we use linear theory of elasticity to develop a new correction to Sneddon's solutions [17] for a conical tip indentation on thin layer, which could be used to improve the evaluation of the viscoelastic properties of flattened cell by nanoindentation. Meanwhile, an AFM-based creep test is performed on human hepatocellular carcinoma (SMMC-7721 cell), being one of the most common cancer types worldwide, and its fullerenol-treated counterpart. The validity of the present model is demonstrated by fitting it to the experimental data. The extracted viscoelastic parameters by our correction model are compared to the values determined by the conventional Sneddon's solutions to verify that the present model could deal with substrate effect. Moreover, the determined viscoelastic properties of normal SMMC-7721 cells are different from their fullerenol-treated counterparts, suggesting that the biomechanical parameters determined by our correction model could also be used as biomarker to evaluate the effects of fullerenol or other anticancer agents on the cells and thus can represent a crucial part of the potential cancer progression. In addition, it is worth noting that measurements of absolute values of viscoelastic modulus of cell prove to be a powerful tool to quantify the effect mutations of intracellular scaffolds (i.e., actin cortex) [18]. The semianalytical dependence of indentation depth on time is given, which is more convenient in practical applications. To the best of our knowledge, the present study represents a first attempt of applying linear theory of elasticity to flattened cell to quantify its viscoelastic properties.

## 2. Theoretical Model

### 2.1. Formulation of Elastic Film Indented by a Rigid Conic Tip

**Figure 1:**
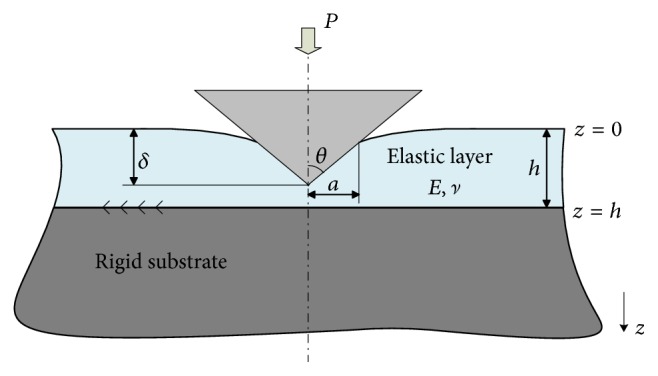
Axisymmetric contact between a frictionless conic and an elastic layer perfected bonded to rigid substrate.

Consider the axisymmetric contact problem of a rigid conic tip on an elastic layer as illustrated in Figure 1. The layer is perfectly bonded to the rigid substrate at the interface *z* = *h* while the contact between the indenter and the film is assumed to be frictionless. The cylindrical coordinate (*r*, *φ*, *z*) is used as shown in Figure 1, where the origin coincides with the overlapping point between the generatrix of the conic and the upper face of undeformed layer. Under these assumptions, the displacement boundary condition consists of

(1a) w=δ−Ψr0≤r≤a,  z=0,

(1b) w=u=00≤r<∞,  z=h

and the stress boundary condition is comprised of

(2a) σz=0r>a,  z=0,

(2b) τrz=00≤r<∞,  z=0,

where *σ*_*z*_ and *τ*_*rz*_ are the normal and tangential stress components, respectively, and* Ψ*(*r*) denotes the axisymmetric shape of the indenter. Since the deformation of the layer subjected to the normal force *P* is axisymmetric, the radial displacement *u* and vertical displacement *w* are independent of the hoop coordinate *φ* and they satisfy the field equations of the linear theory of elasticity [19] for homogeneous, isotropic materials, given as

(3) $$\begin{array}{c} \left(\;1-2\nu \;\right)\nabla^{2}u+\nabla \left(\;\nabla \cdot u\;\right)=0, \end{array}$$

where **u** = (*u*, 0, *w*) is the displacement vector, *ν* denotes Poisson's ratio, and ∇ represents gradient operator.

The solution of the axisymmetric contact problem depicted in Figure 1 could be solved in terms of Papkovich–Neuber solution for the expression of the components of displacement vector

(4a) 2Gur=−∂∂rΦ0r,z+zΦ1r,z,

(4b) 2Guz=−∂∂zΦ0r,z−z∂∂zΦ1r,z+3−4νΦ1r,z

and stress vector

(5a) σz=21−ν∂∂zΦ1r,z−∂2∂z2Φ0r,z−z∂2∂z2·Φ1r,z,

(5b) τrz=∂∂r1−2νΦ1r,z−∂∂zΦ0r,z−z∂∂zΦ1r,z,

where Φ_*i*_  (*i* = 0,1) is harmonic function known as the Boussinesq–Papkovich potential functions [20] and *G* denotes shear modulus. Since the solution of stress and displacement under the mixed boundary conditions has been developed by many studies [21, 22], its detailed derivation procedure is not repeated in this study. Herein, we directly formulate the dependence of indentation force *P* and indentation depth *δ* on the contact radius *a* as

(6a) P=2aEδ1−ν2∫01ωτdτ,

(6b) δ=−π2acot⁡θωc1ωf1,

respectively, where *ω*(*τ*) = *ω*_*f*_(*τ*) + *πaω*_*c*_(*τ*)cot⁡*θ*/2*δ* and *ω*_*c*_(*τ*) and *ω*_*f*_(*τ*) are the solutions of the following Fredholm integral equation of the second kind:

(7a) ωcξ+1π∫τ=01ωcτKτ+ξ+Kτ−ξdτ=−ξ,

(7b) ωfξ+1π∫τ=01ωfτKτ+ξ+Kτ−ξdτ=1,

respectively, in which

(8) Ku=ah·∫α=0∞3−4νe−αshα−α1+α−41−ν2α2+41−ν2+3−4νsh2αcos⁡αauhdα⁡.

**Figure 2:**
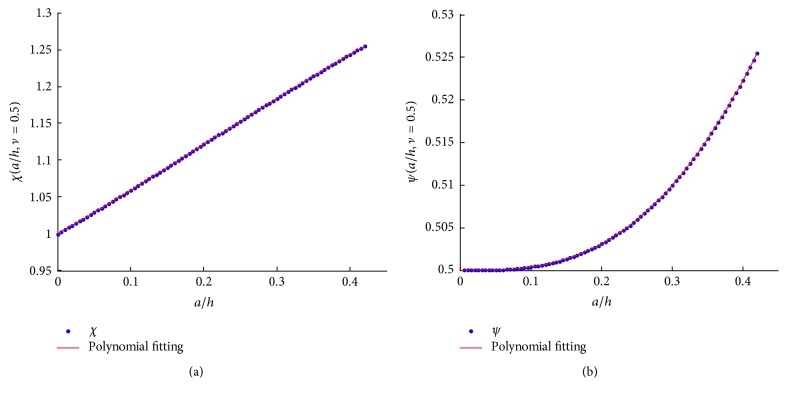
Polynomial fitting results of (a) *χ*(*a*/*h*, *ν* = 0.5) and (b) *ψ*(*a*/*h*, *ν* = 0.5).

If the dimensionless parameters

(9a) κah,ν=∫01ωτdτ,

(9b) χah,ν=−ωc1ωf1,

(9c) ψah,ν=κa/h,νχa/h,ν

are introduced, (6a) and (6b) could be rewritten as

(10a) P=πE1−ν2a2cot⁡θ·ψah,ν,

(10b) δ=πa·cot⁡θ2χa/h,ν,

respectively. The two Fredholm integral equations of the second kind could be solved by numerical method for a given value of* a*/*h* and *ν*. Since biological cell is always treated as incompressible, Poisson's ratio *ν* equals 0.5, and thus the three dimensionless parameters are only dependent on* a*/*h*. After application of numerical techniques to (7a) and (7b), *χ* and *ψ* are solved and fitted by polynomials by least square method, formulated as

(11a) χah=−0.46ah3+0.28ah2+0.57ah+1,

(11b) ψah=−0.26ah4+0.47ah3−0.006ah2+0.0003ah+0.5,

respectively, whose fitting results are plotted in Figure 2. Eliminating *a* in (10a) and (10b) could result in

(12) $$\begin{array}{c} P=\frac{2E\delta^{2}\tan\theta }{\pi \left(1-\nu^{2}\right)}\cdot 2\chi^{2}\psi . \end{array}$$

### 2.2. Viscoelastic Situation

**Figure 3:**
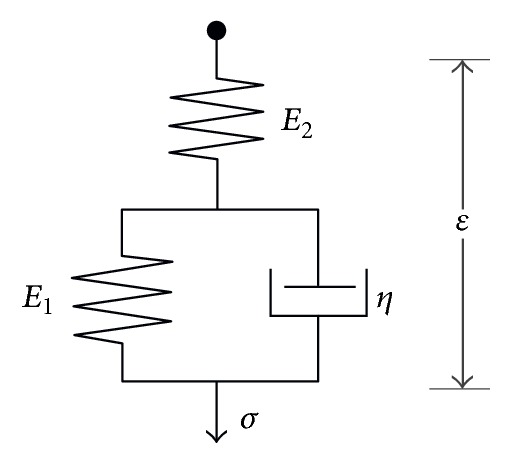
Schematic diagram of standard solid model where a first spring (whose stiffness is *E*_1_) is in parallel with a dashpot and then connected with a second spring (whose stiffness is *E*_2_).

The viscoelastic behavior of materials can be simulated by the* standard solid* [23], which is shown in Figure 3. It is comprised of an elastic spring, which describes an instantaneous elastic deformation, placed in series with a parallel combination of a spring and dashpot, which describes a delayed elastic deformation. The stress *σ* applied on the spring element is proportional to its strain *ε*; that is, *σ* = *Eε*, while the stress on the dashpot element is proportional to the rate of its strain; that is, *σ* = *η* · *dε*/*dt*. The coefficients *E* and *η* denote elastic modulus and viscosity, respectively. For the constitution shown in Figure 3, the corresponding constitutive relation is given as

(13) $$\begin{array}{c} \sigma +\frac{\eta }{E_{1}+E_{2}}\frac{d\sigma }{dt}=\frac{E_{1}E_{2}}{E_{1}+E_{2}}\varepsilon +\frac{\eta E_{2}}{E_{1}+E_{2}}\frac{d\varepsilon }{dt}, \end{array}$$

where *E*_1_ and *E*_2_ denote the two spring constants. If the stress *σ* is a unit Heaviside step function *σ* = *H*(*t*), the corresponding output strain is termed creep compliance *J*(*t*), given as

(14) $$\begin{array}{c} J\left(\;t\;\right)=\frac{1}{E_{2}}+\frac{1-e^{-t/\tau }}{E_{1}}, \end{array}$$

where *τ* = *η*/*E*_1_, termed characteristic retardation time corresponding to the time during which the sample deforms by 1 − *e*^−1^ (or 63.2%) of the total creep deformation. It can be seen from (14) that *J*(0^+^) = 1/*E*_2_ and *J*(*∞*) = 1/*E*_1_ + 1/*E*_2_. Therefore, the* standard solid *model has an instantaneous modulus *E*_0_ = *E*_2_ and equilibrium modulus *E*_*∞*_ = *E*_1_*E*_2_/(*E*_1_ + *E*_2_). It should be pointed out that the* standard solid* model is a relatively universal model and it covers two extreme cases. For example, as *E*_2_ → *∞*, Figure 3 degrades to a spring in parallel with a dashpot (Kelvin model) while as *E*_1_ → 0,* standard solid* model reduces to a spring in series with a dashpot (Maxwell model).

For the viscoelastic situation, both Lee and Radok [24] and Ting [25] offered a general solution to linear viscoelastic Boussinesq problem (an infinite half-space indented by an arbitrary shape of rigid, axisymmetric, and frictionless punch) as long as the contact radius is nondecreasing as mutual approach increases. According to their theory, substituting the elastic modulus in Sneddon's solutions with the modulus-displacement convolution in the time domain leads to the relationship between the contact radius *a* and the applied force *F* as [26, 27]

(15) $$\begin{array}{c} a^{2}\left(\;t\;\right)\psi \left(\;\frac{a}{h}\;\right)=\frac{1-\nu^{2}}{\pi }\tan\theta \cdot J\left(\;t\;\right)*F\left(\;t\;\right), \end{array}$$

where the asterisk denotes convolution; that is,

(16) $$\begin{array}{c} J\left(\;t\;\right)*F\left(\;t\;\right)=\overset{t}{\underset{{\xi =0}^{-}}{\int }}J\left(\;t-\xi \;\right)\frac{d}{d\xi }F\left(\;\xi \;\right)d\xi . \end{array}$$

Performing Laplace transform on both sides of (16) yields

(17) La2tψah=1−ν2πtan⁡θLJtLdFtdt.

If* F*(*t*) is assumed to be a Heaviside step function, one has

(18) $$\begin{array}{c} L\left[\;a^{2}\left(\;t\;\right)\psi \left(\;\frac{a}{h}\;\right)\;\right]=\frac{1-\nu^{2}}{\pi }\tan\theta L\left[\;J\left(\;t\;\right)\;\right]F_{max}. \end{array}$$

Performing inverse Laplace transform on (18) results in

(19) $$\begin{array}{c} {\left[\;\frac{a\left(t\right)}{h}\;\right]}^{2}\psi \left[\;\frac{a\left(t\right)}{h}\;\right]=\frac{1-\nu^{2}}{\pi }J\left(\;t\;\right)\frac{F_{max}}{h^{2}}\cdot \tan\theta . \end{array}$$

On the other hand, the time-dependent indentation *δ*(*t*) and contact radius *a*(*t*) are also related by (10b); that is,

(20) $$\begin{array}{c} \delta \left(\;t\;\right)=\frac{\pi a\left(t\right)\cdot \cot\theta }{2\chi \left[a\left(t\right)/h\right]}. \end{array}$$

Therefore, the dependence of indentation depth *δ*(*t*) on time could be derived by solving (19) and substituting *a*(*t*) into (20), which is ready for fitting the *δ*-*t* curve obtained by experiment.

## 3. Materials and Methods

To validate the present model, AFM-based creep tests have been performed on SMMC-7721 cell.

### 3.1. Cell Preparation

SMMC-7721 cells were revived after being frozen in freezer and were incubated in Roswell Park Memorial Institute- (RPMI-) 1640 media with 10% of fetal bovine serum (FBS) and antibiotics (penicillin-streptomycin solution). The protocol for the culture and fullerenol treatment of SMMC-7721 cells have been described in detail elsewhere [28].

### 3.2. Atomic Force Microscopy

**Figure 4:**
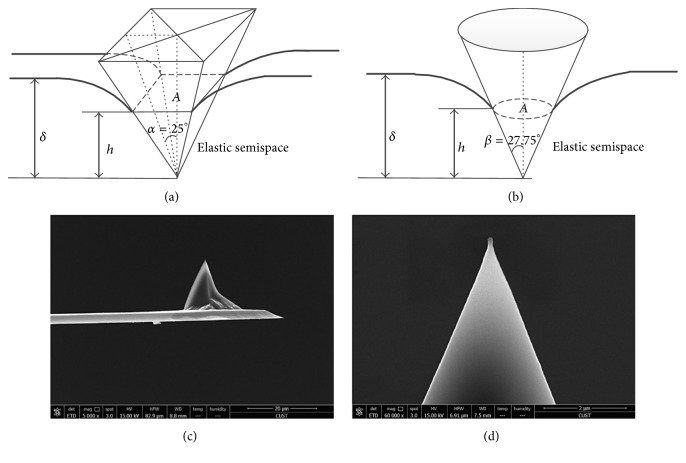
Schematic of a compliant semi-infinite space indented by (a) a square pyramid and (b) a conic indenter. *α* and *β* denote the half-opening angle of the pyramid and conic indenter, respectively.

The module of the AFM employed in this study is JPK NanoWizards 3 BioScience (Berlin, Germany), and it is mounted on an inverted optical microscope (Olympus IX71; Tokyo, Japan), allowing the AFM and optical microscope imaging simultaneously. The criterion for cantilever selection is that the compliance of the cantilever should be within the range of the sample compliance. For very soft and delicate cells, the softest cantilevers are available with spring constants ranging from 0.01 to 0.06 N/m (JPK Application Note). Before indentation, the spring constant of the AFM cantilever was calibrated. A silicon nitride cantilever, whose spring constant is 0.059 N/m after calibration, was used for cell-tip indentation in this work. The probe is a square pyramid tip with a half-opening angle of *α* = 25° (half-angle to face), and its radius and height are 10 nm and 4 *μ*m, respectively, as can be seen in Figures 4(c) and 4(d).

Figure 4(a) shows schematically that the displacement of a pyramid tip along a distance *δ* inside a half-space material creates a tip-material contact area, which is determined by the contact depth *h*. Since the AFM cantilever tip is a pyramid, the projection area *A* of the tip-sample contact surface is not circular, that is, not axisymmetric. However, numerical analysis [29, 30] indicates that Figure 4(a) could be approximated by the contact between a conic indenter and substrate material as illustrated in Figure 4(b) with a negligible error of 0.012, as long as the conic gives the same projected area-to-depth ratio* A*/*h* as that of pyramid. In this regard, the half-opening angle *β* of the conic equals 27.75° in order to retain the same area-to-depth ratio of pyramid shown in Figure 4(a).

### 3.3. Loading Method

**Figure 5:**
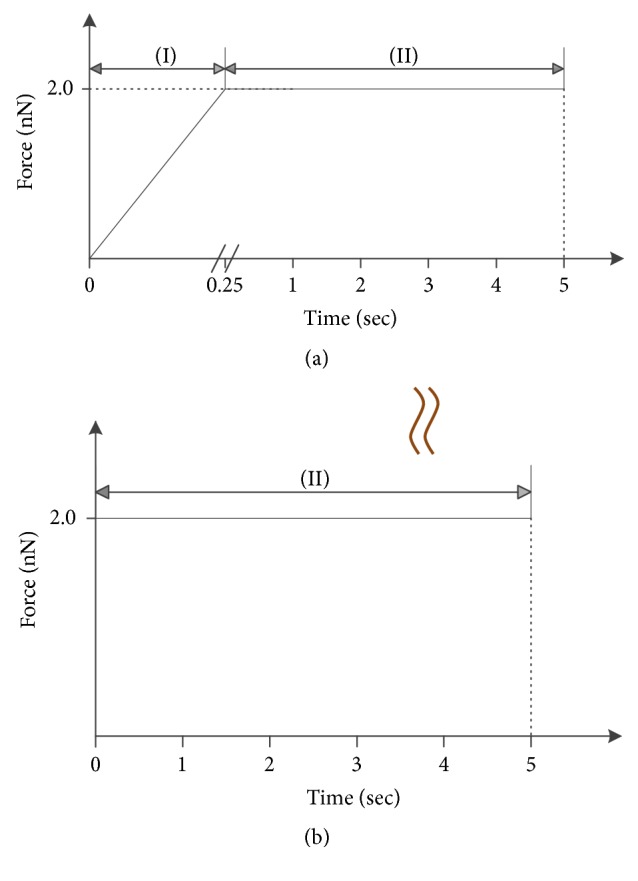
Schematic of the AFM indentation force versus time (a) and its approximation (b) by Heaviside step function.

The determination of viscoelastic properties of material is commonly realized by the creep response to a prescribed load. The loading method of indentation force illustrated in Figure 5 is to realize the creep test on single cells.  Figure 5(a) depicts the factual loading history, which could be approximated by a Heaviside step function as shown in Figure 5(b), as long as the loading period (stage (I)) is smaller than one-tenth of that of dwelling period (stage (II)) [11]. In the present AFM-based creep test, the creep tests were conducted by constant force delay mode where the force reaches its maximum value (2 nN) within 0.25 seconds and resides at the peak value for 5 seconds.

## 4. Results and Discussions

### 4.1. Cell Topography Analysis

**Figure 6:**
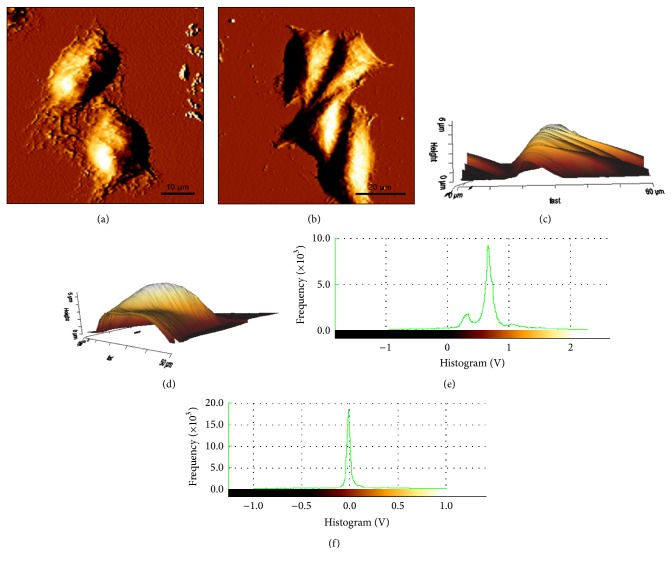
(a) and (b) represent AFM deflection image of control and treated cells, respectively. (c) and (d) denote the 3D distribution of cell height for control and treated cells, respectively. (e) and (f) are the corresponding statistics of cell height value for control and treated cells, respectively.

**Figure 7:**
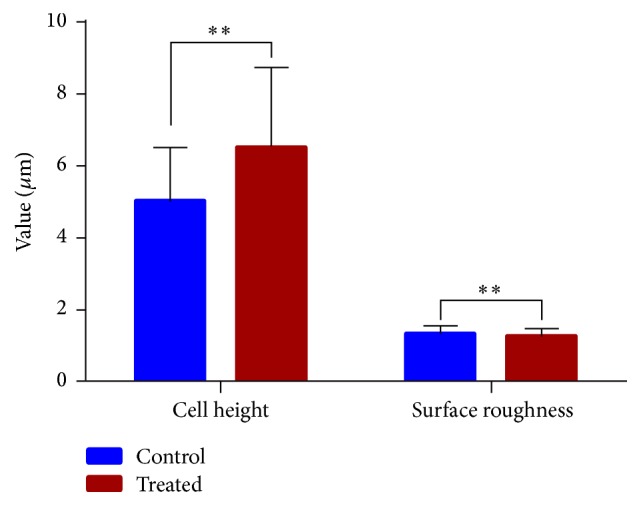
The statistics results of cell height and surface roughness. Data are expressed as mean ± SEM of more than 30 cells from 3 separate experiments, where key significance values are shown, ^*∗∗*^*p* < 0.01.

Priority to creep test, the contact mode of AFM was used for topography imaging of the cells. The AFM deflection images of both control (Figure 6(a)) and treated cell (Figure 6(b)) were obtained by the AFM contact imaging mode in real time. The majority of control cell shapes are polygonal (Figure 6(a)) while, after being treated by fullerenol, the SMMC-7721 cell exhibits a significant change from polygon to shuttle as shown in Figure 6(b). In addition, the AFM deflection imaging can also enable us to investigate the height distribution of individual cells. The 3D view of cell topography (Figures 6(c) and 6(d)) indicates that both control and treated cells spread above the substrate, which is further confirmed by the histogram (Figures 6(e) and 6(f)) of pixel value where the narrow range suggests that the cell is fairly flat. The statistical analyses of the cell height and surface roughness of the control and treated cells are shown in Figure 7. Significant increase in the mean height after fullerenol treatment could be observed while there are no conspicuous variations in the surface roughness between the two types of cells.

### 4.2. Analysis of Creep Test Curves

**Figure 8:**
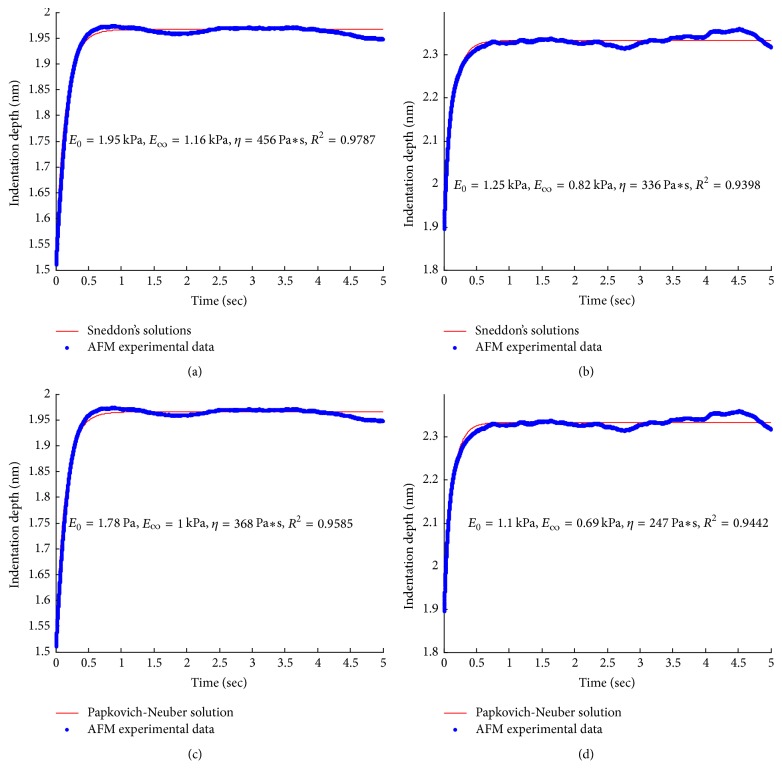
Fitting results of control cells ((a) and (c)) and treated cells ((b) and (d)) by the two models.

Although the elastic modulus is frequently used to characterize the mechanical properties of biological cells, it does not present a complete description. It can be seen from the blue bold curves in Figure 8 that the cells exhibit a time-dependent deformation under the invariant indentation force; that is, the cells creep. Therefore, it is more appropriate to treat the cell as viscoelastic. The mechanical response of the cell to the applied force ranges on a time scale of several seconds, which is very slow compared with the loading time. Therefore, the mechanical response of the cell is divided into two components: an instantaneous, elastic response and a delayed elastic response due to creep deformation. In this study,* standard solid* model of viscoelasticity theory is used to describe the mechanical response of the cell, which is characterized by three parameters: instantaneous modulus *E*_0_, equilibrium modulus* E*_∞_, and viscosity *η*, as introduced by (13) and Figure 3. Since SMMC-7721 cell spreads like film as analyzed in Section 4.1, the present contact model developed in Section 2 is justified for fitting process, in which the local thickness of the indented point was estimated by the AFM deflection imaging function as mentioned in Section 4.1. For the purpose of comparison, Sneddon's solutions are also used for fitting where the cell is treated as semi-infinite space. We find that the fits of these two models to the creep deformation data are very good regardless of the cell type, as can be seen in Figure 8, with coefficient of determination close to one (*R*^2^ ≥ 0.93).

### 4.3. Cell Viscoelastic Properties

**Figure 9:**
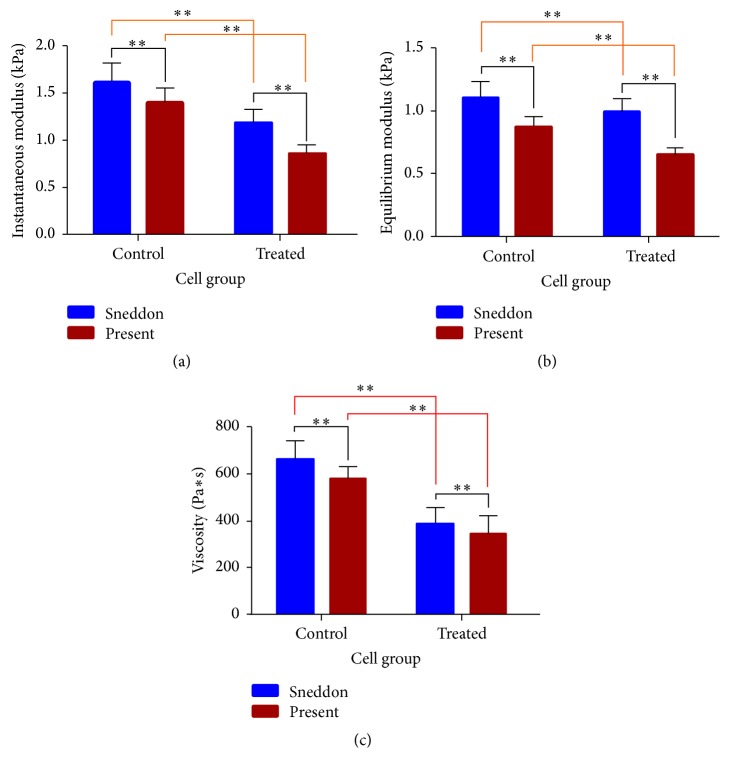
The statistics results of (a) *E*_0_, (b)* E*_∞_, and (c)*η*. Data are expressed as mean ± SEM of more than 30 cells from 3 separate experiments, where key significance values are shown, ^*∗∗*^*p* < 0.01.

The viscoelastic parameters of control and treated cells were determined according to Sneddon's solutions and the present model, and their mean values are presented in Figure 9. In the present model, the value of cell thickness is determined by the AFM deflection imaging. It can be seen that the three parameters determined by the present model are lower than that determined by Sneddon's solutions, regardless of the cell type, which indicates that the present model could alleviate the overestimation of biomechanical properties by Sneddon's solutions.

From Figure 9, it could be also seen that the average elastic modulus and viscosity of the treated cells show a diminishing trend compared to those control cells, regardless of whichever model adopted. Concretely, control cells have significantly higher (*p* < 0.01) instantaneous modulus and viscosity than the instantaneous modulus and viscosity of treated cells, while the equilibrium modulus of control cell is slightly higher that its treated counterpart. Previous studies have already reported that both elasticity and viscosity are heavily impacted by the levels and organization state of actin cortex [31]. Since actin cortex is transformed into actin aggregates and distributed irregularly within the cells after being treated by fullerenol [32], we infer that this transform of actin cortex induces variation of the viscoelastic parameters of SMMC-7721 cells.

In our study, we treat the cell as a homogeneous material and thus present a global equivalent quantification of viscoelastic properties of the cell. We admit that the assumption of homogeneity is a limitation in our present work, and inhomogeneous model would present more details. For example, Feneberg et al. [33] measured shear elasticity of cell envelops using magnetic tweezer technique, which is important in terms of providing insight into the structure of cell envelops or cytoplasm.

### 4.4. Validation and Comparison

**Figure 10:**
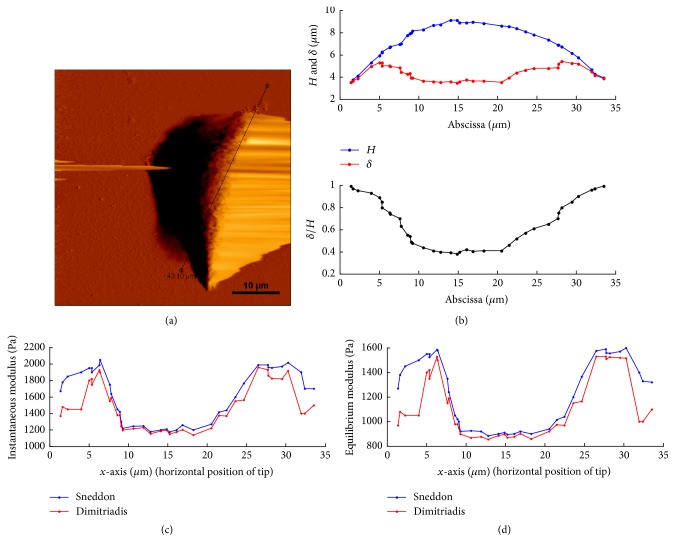
(a) Deflection image of single control cell with the scanning range of 55 × 55 *μ*m. (b) Cell height profile, indentation depth, and*δ*/*H* variation along cut path. (c) and (d) denote elastic moduli variation along cut path.

In order to further validate the capability of the present model in alleviating the substrate effect, we present a test of it on different height area of cell elaborated as follows. As shown in Figure 10(a), we select an arbitrary intersecting surface and plot the variation of cell height along the cut path. Creep tests are performed along the path and the indentation depth-time curves are fitted by both models. The variation of the instantaneous modulus (*E*_0_) and the equilibrium modulus *E*_*∞*_ along the cutting path is plotted in Figures 10(c) and 10(d), respectively. At the nucleus region (10 < *x* < 20 *μ*m), the determined elastic moduli exhibit uniformity, indicating material homogeneity in this area. In the region around the nucleus (5 < *x* < 10 *μ*m and  22 < *x* < 30 *μ*m), there exists actin filaments network which plays a key role in cellular mechanical stability, and therefore we observe increase of elastic modulus in this area. At the margin of the cell (0 < *x* < 5 *μ*m and  30 < *x* < 34 *μ*m), the elastic modulus decreases since the density of actin filaments declines in this area. In all regions, Sneddon's solutions result in higher elastic modulus compared to the present model. In the nucleus region where the local thickness is high, the ratio *δ*/*H* is low, and thus one gets low value of the correction factor in (12), resulting in nearly equal elastic modulus predicted by the two models. The substrate effect becomes significant at the margin area, where the multiplicative factor dominates in (12).

## 5. Conclusions

In this paper, we first introduce the present model based on the contact mechanics of thin film, and this model underlies the interpretation of flattened cell subjected to AFM indentation. The present model relieves the major assumption of semi-infinite space of classic Sneddon's solutions to account for the realistic morphology of spread cells. Afterwards, the model is extended to viscoelastic constitution to reflect cell's viscoelastic nature. The AFM-based creep test was conducted to validate the present model. The topography imaging of SMMC-7721 cell confirms that cells exhibit flattened morphology which justifies the application of the present model. The fitting results have shown that the present model can not only describe very well the creep behavior of the SMMC-7721 cell, but also avoid the overestimation of elastic and viscosity properties of thin film due to substrate effect. Hereupon, we account for the suppression of overestimation by the present model in terms of correction factor. In addition, the present model could identify the variations of the SMMC-7721 cell and its fullerenol-treated counterpart in terms of the extracted viscoelastic parameters, which reveals its instructive significance in understanding fullerenol-induced effect on the viscoelastic properties of cancerous cells and the potential in anticancer drug in terms of fullerenol application.
